# Modifications of Default-Mode Network Connectivity in Patients with Cerebral Glioma

**DOI:** 10.1371/journal.pone.0040231

**Published:** 2012-07-09

**Authors:** Roberto Esposito, Peter A. Mattei, Chiara Briganti, Gian Luca Romani, Armando Tartaro, Massimo Caulo

**Affiliations:** 1 Institute for Advanced Biomedical Technologies, “G. D’Annunzio” University Foundation, Chieti, Italy; 2 Department of Neuroscience and Imaging, University of “G. D’Annunzio,” Chieti, Italy; Hangzhou Normal University, China

## Abstract

**Purpose:**

The aim of the study was to evaluate connectivity modifications in the Default Mode Network (DMN) in patients with cerebral glioma, and to correlate these modifications to tumor characteristics.

**Methods:**

Twenty-four patients with a left-hemisphere cerebral tumor (14 grade II and 10 grade IV gliomas) and 14 healthy age-matched right-hand volunteers were enrolled in the study. Subjects underwent fMRI while performing language tasks for presurgical mapping. Data was analyzed with independent component analysis in order to identify the DMN. DMN group maps were produced by random-effect analysis (p<0.001, FDR-corrected). An analysis of variance across the three groups (p<0.05) and post-hoc t-test contrasts between pairs of groups were calculated (p<0.05, FDR-corrected).

**Results:**

All three groups showed typical DMN areas. However, reduced DMN connectivity was detected in tumor patients with respect to controls. A significantly increased and reduced integration of DMN areas was observed in the hippocampal and prefrontal regions, respectively. Modifications were closely related to tumor grading. Moreover, the DMN lateralized to the hemisphere contralateral to tumor in the low-grade, but not in the high-grade tumor patients.

**Conclusion:**

Modifications of DMN connectivity were induced by gliomas and differed for high and low grade tumors.

## Introduction

Functional neuroimaging studies typically focused on task-related activations; i.e., increases in brain activity observed by subtracting a reference state from an activated state. Interest in areas of the brain with decreased activation during task performance, commonly referred to as deactivation, is increasing [1]. While specific areas of brain activation were strongly correlated to the task performed, deactivated areas were reported to be substantially task-independent and included medial prefrontal cortex, posterior cingulate cortex, medial parietal cortex, inferior temporal cortex and hippocampal regions. This set of brain areas was implicated in attending to external and internal stimuli, as well as self-referential and reflective activity [1], [2]. The fact that, even at rest, these regions show high metabolism and prominent long-range coherent activity suggested that they comprise an organized functional network, namely the Default Mode Network (DMN) [3].

The presence of functional connectivity based on temporal correlations across distant brain areas is the common criterion for defining brain networks [4]. Functional connectivity studies showed that the DMN is not the only network with coherent activity; i.e., the brain is organized in a set of widely distributed networks, commonly modulated during active behavioral tasks [5].

Given this novel view of the brain’s functional architecture it was hypothesized that focal injury disrupts synchronization between the site of damage and other connected regions within the network. Also changes in the state of one network may affect the dynamic state of other connected networks [6]. The results of functional connectivity studies on clinical populations strongly support this hypothesis [7]–[9]. Furthermore, brain plasticity processes associated with function recovery following a focal brain lesion involve not only local brain areas, but require the reorganization of all brain networks [6], [10].

In patients with cerebral tumor, plasticity plays an important role in filling in for damaged areas (language, motor function, sight, etc.), thus preserving primary functions and ensuring a good quality of life. For example, several studies of language plasticity documented that the presence of lesions distant to language areas generated a recruitment of remote brain areas in the ipsilateral or contralateral hemisphere to the lesion, especially in slow-growing lesions like low-grade gliomas [11]–[13]. Furthermore, studies in brain tumor patients reported cognitive dysfunction in multiple domains (e.g., reduction of attention capacity, depression, and working memory problems) [14], [15], which is not easily attributable to local injury, thus suggesting a global impairment of neural networks induced by tumors [16], [17].

Evidence supporting the hypothesis that DMN connectivity is a biomarker in brain physiology and pathology is increasing. Studies reported the relevance of DMN in understanding numerous brain diseases (including schizophrenia, autism and Alzheimer’s disease), indicating a correlation between specific pathological conditions and observed modifications of DMN [18], [19]. Therefore further evaluation of temporal and spatial modulation of DMN as a biomarker of other brain diseases is required.

The aim of the present study was to compare the DMN of healthy controls with patients with low-grade and high-grade glioma, in order to evaluate correlations between DMN modifications and tumor grading.

## Materials and Methods

### Study Population

All participants gave written informed consent prior to enrollment in the study, which was approved by the Comitato Etico dell'Università degli Studi “G. d'Annunzio” di Chieti-Pescara. All procedures were performed according to the principles expressed in the Declaration of Helsinki. We retrospectively evaluated 24 non-aphasic patients with non-operated left frontal and/or temporo-parietal lobe brain glioma (histologically confirmed as 10 WHO grade II (low-grade) and 14 WHO grade IV (high-grade) gliomas) [20] and 14 age-matched healthy volunteers (Table 1). Patients and controls had normal hearing and vision, and were right-handed as determined by the Edinburgh Handedness Inventory test with a laterality quotient >80 [21]. Tumor volume was obtained by an expert neuro-radiologist with 10 years of experience who manually segmented the tumors using 3D T1-weighted high-resolution images, as well as pre- and post-Gadolinium images.

**Table 1:** Characteristics of patients with brain tumors.

Gender/Age	Diagnosis (Histology)	Grade	Location (left hemisphere)	Edema	Volume (mm^3^)
M/34	Pleomorphic Xanthoastrocytoma	Low	Temporo-Parietal	–	3118
M/37	Astrocytoma	Low	Temporal	+	47900
M/41	Ganglioglioma	Low	Frontal	–	11000
F/42	Oligodendroglioma	Low	Fronto-Temporal	+	37927
M/38	Astrocytoma	Low	Temporal	+	63681
M/30	Oligoastrocytoma	Low	Temporal	–	25446
F/56	Oligodendroglioma	Low	Frontal	–	7000
M/37	Oligoastrocytoma	Low	Fronto-Temporal	–	40254
F/59	Glioma	Low	Frontal	–	8433
M/55	Glioma	Low	Temporal	–	3492
F/67	Astrocytoma (III grade)	High	Frontal	–	3126
F/65	Astrocytoma (III grade)	High	Temporal	–	11197
M/38	Astrocytoma (III grade)	High	Frontal	–	79503
M/33	Astrocytoma (III grade)	High	Temporo-Parieto-Occipital	+	141589
M/46	Anaplastic Astrocytoma	High	Temporo-Parietal	+	47333
M/44	Glioblastoma multiforme	High	Temporal	–	48138
M/63	High-grade Glioma	High	Fronto-Temporo-Parietal	+	48688
M/57	High-grade Glioma	High	Temporo-Parietal	–	73341
F/58	Glioblastoma multiforme	High	Temporal	–	9724
M/66	Anaplastic Oligoastrocytoma	High	Fronto-Parietal	–	31990
F/48	Anaplastic Astrocytoma	High	Parietal	+	79422
M/66	Anaplastic Astrocytoma	High	Parietal	–	27300
F/27	Astrocytoma	High	Frontal	+	22199
F/42	Glioblastoma multiforme	High	Temporo-Parietal	–	11764

**Figure 1:**
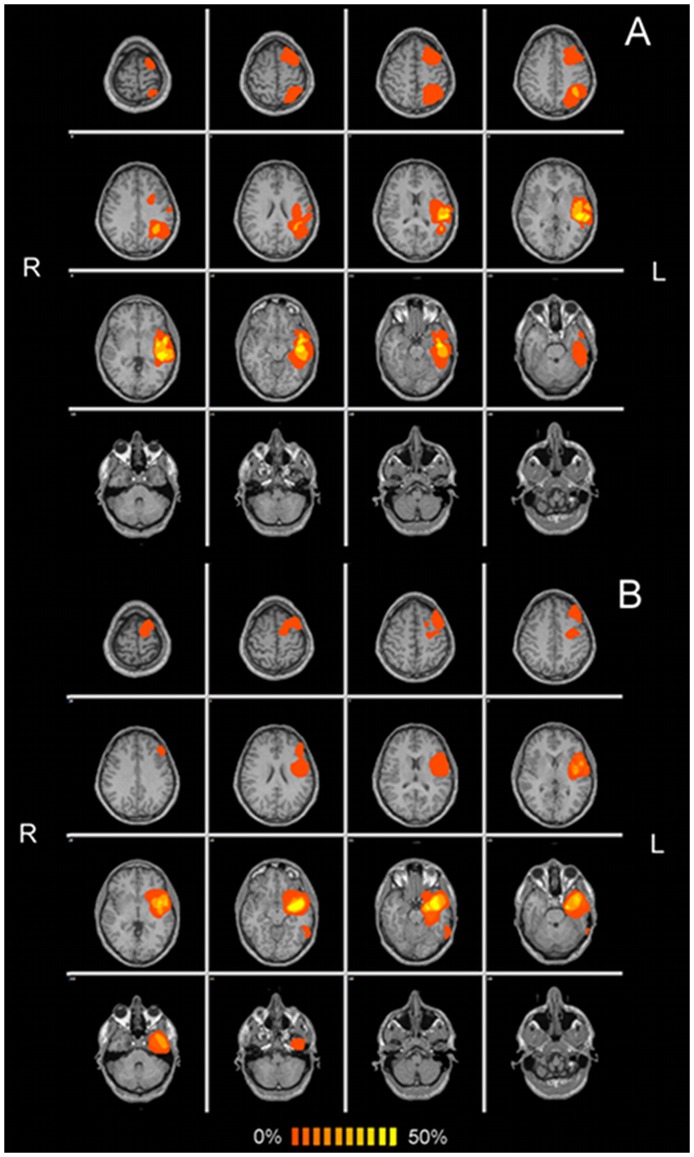
Frequency map of the spatial distribution of high (A) and low (B) grade tumors superimposed on a standard anatomical template, in radiological convention. An overlap between tumor volume and DMN was present in 4 patients: one with low grade (overlap of 230 mm^3^) and three with high grade (maximum overlap of 10930 mm^3^) tumors.

Position and size of tumors in the two groups are shown in Figure 1. In no cases did the tumor determine either a considerable shift of the hemispherical midline or an anatomical deformation of the AC or PC. No statistical differences were observed in the distances between AC and lateral points of controls and patients indicating that tumors did not compromise spatial normalization. An overlap between tumor volume and DMN was present in 4 patients: one with low grade (overlap of 230 mm^3^) and three with high grade (maximum overlap of 10930 mm^3^) tumors.

### Experimental task

Each subject was asked to silently perform an orthographically-cued, block-designed, verb-generation task, for the pre-surgical mapping of language areas. The task was divided into four task periods (30s each) intermixed with five rest periods (20s each). During each task period, subjects were required to think for 2s of pronouncing one or more verbs associated with a noun, presented at the center of the screen for 1s. During rest periods, subjects were instructed to relax while fixating a central cross. Visual stimuli were presented using E-Prime v.1.1 (Psychology Software Tools) projected via an LCD projector and viewed with a mirror placed above the subject’s head.

### fMRI Acquisition

BOLD functional imaging was performed with a Siemens Magnetom Vision (Siemens, Erlangen. Germany) 1.5 T scanner, using T2*-weighted echo planar imaging free induction decay sequences with the following parameters: TE 60 ms, matrix size 64×64, FOV 256 mm, in-plane voxel size 4×4 mm, flip angle 90°, slice thickness 4 mm and no gap. Functional volumes consisted of 22 transaxial slices, acquired with a volume TR of 3000 ms. Structural images were acquired at the end of the two fMRI runs via a 3D MPRAGE sequence with the following parameters: sagittal, matrix 256×256, FOV 256 mm, slice thickness 1 mm, no gap, in-plane voxel size 1×1 mm, flip angle 12°, TR = 9.7 ms and TE = 4 ms.

### fMRI Analysis

Brain Voyager Q× 1.9 (Brain Innovation, Maastricht, Netherlands) was used for image data preparation and processing. Functional image time-series were first corrected for the differences in slice acquisition time, detrended, realigned with the structural images and warped (six-point non affine registration) into standard Talairach anatomical space.

DMN maps for each dataset were generated by means of ICA; a data-driven method which decomposes the BOLD time-series into a set of independent spatio-temporal patterns (ICs). This method was chosen over a seed based method in order to avoid errors that could have arisen from distortion of normal anatomy induced by tumors. Thirty ICs were estimated by means of the FastICA algorithm [22], with a deflation approach and *tanh* non-linearity [23], [24]. Each IC consisted of a waveform and a spatial map: the waveform corresponded to the time-course of the specific pattern. The intensity of this activity across voxels was expressed by the associated spatial map. The intensity values in each map were scaled to z-scores [2], [25], and thresholded at |z|>2 for visualization purposes. After the exclusion of artifactual ICs [26], the IC showing the largest and most significant spatial correlation with DMN template, obtained in a previous study of resting-state conducted on healthy volunteers [2] was automatically selected by the software. This approach is in line with previous clinical studies on DMN, and assumes that there is a canonical spatial pattern to DMN, which allows it to be reliably detected at the single-subject level using a template-matching procedure [25]. Before statistical analysis, we inverted the polarity of the time-course and spatial map of the DMN ICs with negative correlation between the time-course and expected response to the task. The latter was obtained by convolving a boxcar waveform with an empirically-founded hemodynamic response function (HRF) [27].

After DMN detection, its spatio-temporal properties were characterized. The regressor correlation (RC) was measured by means of Pearson’s correlation coefficient between the DMN time-course and the HRF-convolved boxcar regressor. The lateralization index (LI) was calculated on the basis of the number of active voxels in the left (Vl) and right (Vr) hemisphere with the formula: LI = (Vl-Vr)/(Vl+Vr) [28]. The statistical threshold for ICA was determined as the median of the values previously reported over which the LI did not vary significantly [29].

### Statistical Analysis

For the healthy controls, low-grade and high-grade glioma patients, group-level significance maps were created by combining results across subjects by using a random effects analysis on the DMN spatial maps (p<0.001 corrected with FDR). We assessed the differences between the DMN maps corresponding to the three groups, considering the cluster size and peak t-score. In addition, to evaluate which cerebral areas were characterized by a change in the DMN pattern related to tumor grading, a one-way analysis of variance (ANOVA) was performed across the three groups (p<0.05). Post-hoc t-test contrasts between group pairs were also calculated by t-tests (p<0.05, FDR-corrected). With the aim of detecting specific DMN features related to the presence of a brain glioma, the same statistical analysis was applied not only to the DMN maps, but also to the RC and the LI values.

## Results

### Default Mode Network

The DMNs, along with corresponding time-course, were reliably extracted from each individual fMRI dataset. We observed deactivation of the DMN during task performance, and activation in rest periods in all DMN time-course and spatial maps. However, at the group level, the correspondence of the DMN IC time-course with the expected response to the task was stronger in healthy subjects (|r| = 0.44, p<0.001) than in tumor patients (|r| = 0.25, p = 0.008). The DMN pattern of the healthy control included the bilateral inferior parietal lobule, posterior cingulate/precuneus, and medial frontal cortex, all typical DMN areas. Interestingly, the DMN of tumor patients showed a reorganization of DMN areas, likely induced by brain lesion in the left posterior parietal region. In these cases, we not only found a reduced DMN integration of the left inferior parietal lobule, but also the absence of connectivity with the right inferior parietal lobule. Furthermore, an increase of connectivity was present in the anterior-medial portion of the posterior cingulate.

**Figure 2:**
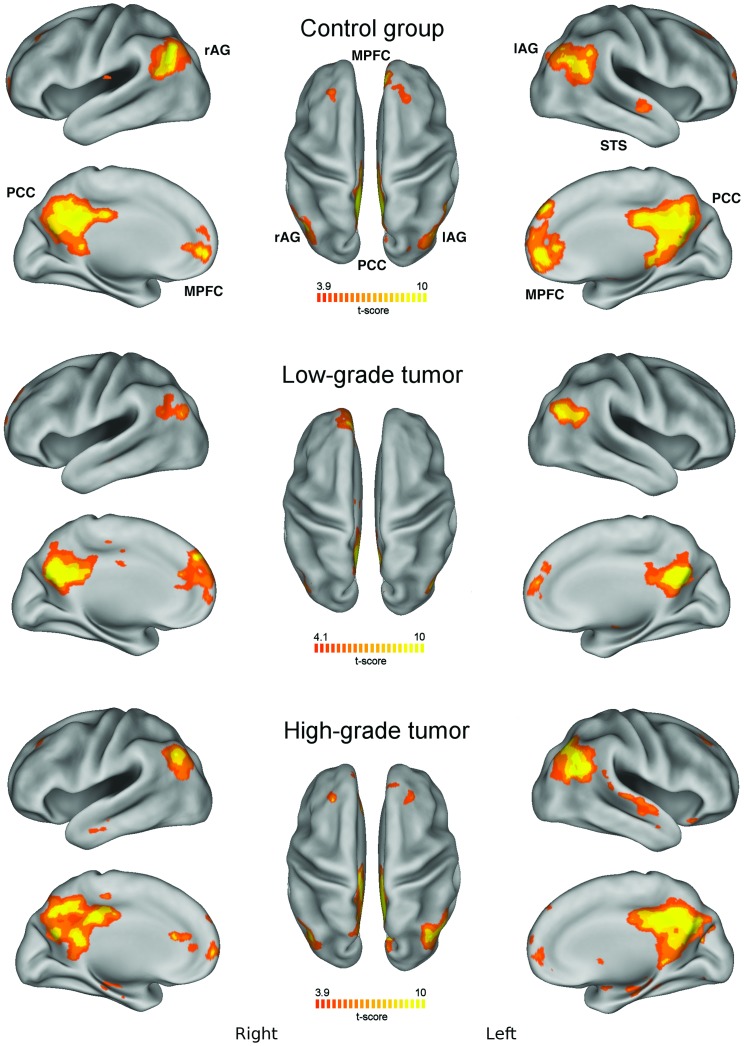
Group-level default mode network (DMN) maps for healthy controls, patients with low-grade and high-grade glioma (p<0.01 corrected with false discovery rate, FDR). For each of the three maps: (Left) Lateral and medial views of the left hemisphere. (Center) Dorsal view. (Right) Lateral and medial views of the right hemisphere. DMN areas are labeled in the control group images (MPFC: medial prefrontal cortex; PCC: posterior cingulate cortex; lAG and rAG: left and right angular gyrus; STS: superior temporal sulcus). The patterns presented by controls and high-grade gliomas have similarities that are not present in low-grade gliomas.

**Figure 3:**
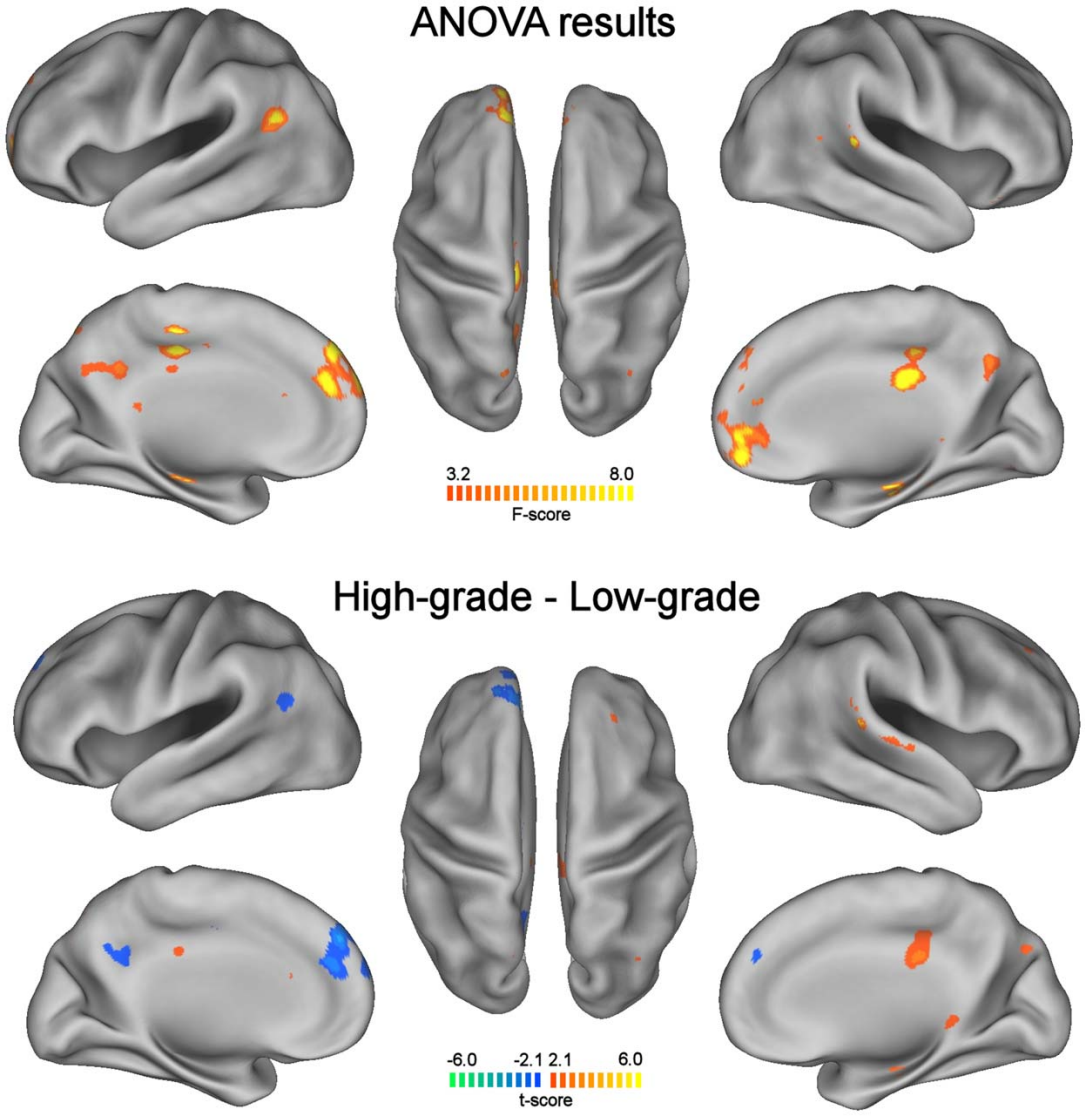
Differences in the DMN for healthy controls, patients with low-grade and high-grade glioma. One-way ANOVA map illustrates the brain areas that show significant changes across the three groups (p<0.05). A t-test contrast map illustrates the brain areas that show significant differences between high-grade and low-grade tumors (p<0.05). A t-test between controls and high-grade tumors did not show significant differences (not presented). For each of the two maps: (Left) Lateral and medial views of the left hemisphere. (Center) Dorsal view. (Right) Lateral and medial views of the right hemisphere.

**Figure 4:**
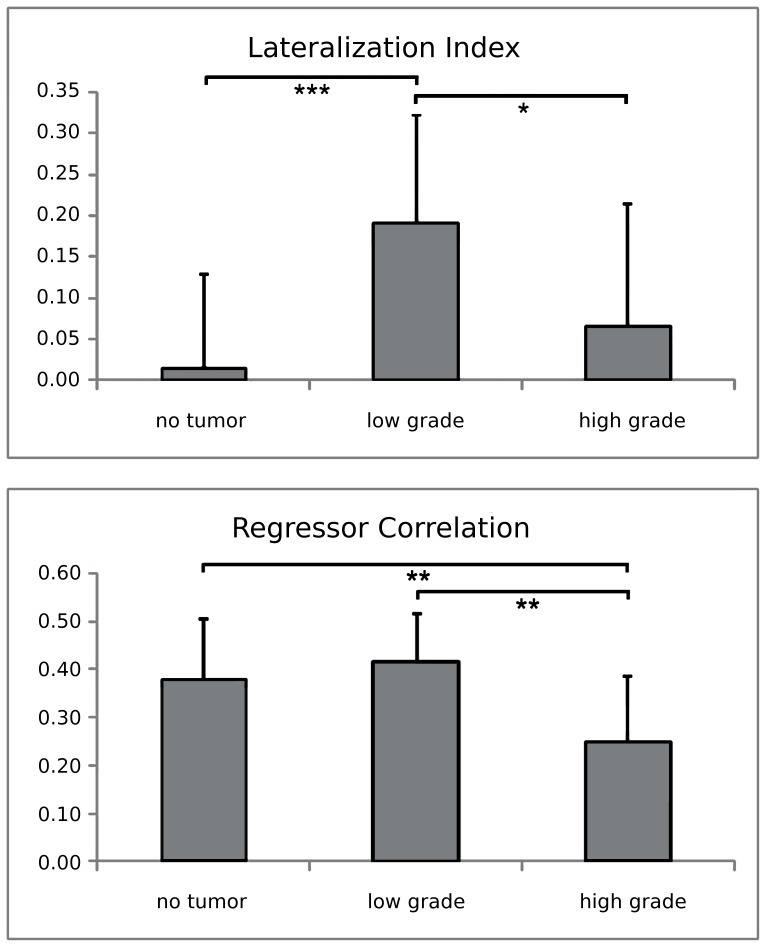
Regressor correlation (RC) and laterality index (LI) of the DMN, for controls and patients with low and high tumor grading. Statistical differences between groups are marked with stars (* = p<0.05, ** = p<0.01, *** = p<0.001).

After group-level analysis, we observed that typical DMN areas were present in all three groups of subjects (p<0.001, FDR-corrected). In the tumor patient groups, the DMN consistently included areas in the left and right hemispheres, despite the fact that all lesions were localized in the left hemisphere. Nonetheless, a larger correspondence of the DMN spatial pattern was qualitatively observed between controls and high-grade tumor patients, whereas larger differences were detected in low-grade tumor patients (Figure 2). A fundamental difference between controls and tumor patients was a general reduced DMN connectivity within cortical areas and an increased connectivity with the hippocampus. This result was statistically significant when evaluated with a one-way ANOVA analysis (p<0.05), indicating a significant modulation of connectivity across the three groups in a large set of DMN areas (Figure 3). By directly comparing the spatial patterns for the high-grade and low grade tumor patients, we found an increased integration of the hippocampus and the posterior cingulate within the DMN, and a reduced integration of the left inferior parietal lobule and the left medial prefrontal cortex (Figure 3). This result suggested the possibility that the DMN could be more right-lateralized in the low-grade than in the high-grade patients. We statistically evaluated this through the analysis of LIs (Figure 4). The DMN, which is typically not lateralized in normal subjects (LI = 0.02±0.11), showed a significant increase of lateralization (p<0.001) in low-grade tumor patients (LI = 0.19±0.13), but no significant difference (p = 0.15) with high-grade tumor patients (LI = 0.06±0.15). Furthermore, the difference in DMN lateralization detected qualitatively between the two patient groups was confirmed by the LI analysis, with a significantly larger LI in low-grade than in high-grade patients (p = 0.02).

### Time-course Analysis

To rule out the possibility that the differences observed in the DMN spatial patterns may be partially related to the difference in the temporal dynamics within the DMN, we also analyzed the correlation of the DMN time-course with the task regressor, for each group separately (Figure 4). In this case, no statistical difference (p = 0.28) was found between the healthy controls (RC = 0.38±0.16) and the low-grade tumor patients (RC = 0.41±0.13). Conversely, a significantly decreased correlation was found in the high-grade tumor patients (RC = 0.25±0.14) with respect to both controls (p = 0.009) and low-grade tumor patients (p = 0.005).

### Tumor-DMN Overlap

Evaluation of the degree of overlap between tumors and components of DMN and the extent of disruption of the DMN was not possible due to the reduced number of patients in which direct overlap was observed.

## Discussion

The aim of this work was to explore the modifications in DMN in patients with cerebral glioma of the left hemisphere by analyzing functional Magnetic Resonance (fMRI) with a data driven method. Analysis focused on the DMN, a specific neural network which is known to be altered in several neurological diseases [19].

### DMN in Brain Tumor Patients

DMN activity is related to active internal mental processes and is most active during passive cognitive states, when thoughts are directed toward internal channels [3]. Based on an increasing number of patient studies reporting a DMN disruption, Buckner and coworkers [1] consistently supported the notion of a relationship between an impaired connectivity within the DMN and cognitive dysfunctions in neurological conditions, particularly in autism, schizophrenia ad Alzheimer’s disease [30]–[32]. To the best of our knowledge, fMRI studies that investigate the DMN alterations in patients with cerebral glioma are lacking. As already demonstrated with magnetoencephalography [33] and diffusion tensor imaging [34]–[35], we found brain plasticity phenomena in regions near and distant to tumors. Our findings were also in line with fMRI studies of patients with stroke and traumatic brain injury; pathologies that presented a reduction of the flow of information between the damaged areas and other functionally connected regions [7], [36]. The inter-and intra-hemispheric functional connectivity between two intact and distinct regions may be compromised in case of damage of other directly or indirectly functionally-connected regions [8], [37], [38]. We found a global reduction of DMN connectivity, which typically included the bilateral inferior parietal lobule, posterior cingulate/precuneus, and medial frontal cortex. This result agrees with the model proposed by Alstott and colleagues, for which the effect of a lesion in the brain is not local, but affects the functional coupling of distant brain regions [6]. We specifically observed a reduced DMN connectivity in the medial prefrontal cortex and bilateral inferior parietal lobule, and an increased connectivity in the hippocampus and anterior-medial portion of the posterior cingulate. The alterations of connectivity in intra- and peritumoral cortical region may explain cognitive dysfunction, such as attention deficits, reduced psychomotor speed and deficit working memory. These symptoms are generally ascribed to the tumor itself, presence of epilepsy and pharmacological treatment, but might be related also to the disruption of connectivity between otherwise intact cortical regions and regions encompassing the neoplasia. In this retrospective study on patients with brain gliomas in the left hemisphere, pre-operative work-up only included the neuropsychological evaluation of language, limiting the possibility to correlate connectivity data to behavior.

### Differences Between Low-grade and High-grade Tumors

In our study we investigated the possible modifications induced by brain tumors on the normal DMN connectivity pattern, and we specifically tested whether the tumor grade influences cerebral plasticity in DMN conformation. We limited the selection of subjects to those who had either WHO grade II or IV tumors in order to avoid low-grade neoplasms that subsequently convert to high grade. As a main result, the DMN resulted lateralized in the right hemisphere (non-dominant) contralateral to the tumor. This difference was statistically significant in the group of low-grade tumor patients. In agreement with the literature on brain plasticity, changes in fMRI activity in patients with cerebral glioma cannot be fully understood without considering the temporal evolution of the pathology. The process of brain plasticity should be more effective in slow-growing lesions like low grade gliomas; thus explaining the large correspondence of the DMN spatial pattern found in our results between controls and high grade tumor patients and not with low grade. The opposite would be expected given the greater overlap of the DMN with high grade tumors. The lack of aggressive growth of low-grade gliomas may permit a more marked plastic reorganization of neural networks (Figure 2). By comparing the DMN spatial maps in the low-grade tumor group with the high-grade tumor group, we also found a significant increase of DMN connectivity in the hippocampal regions. This result might be directly related to the task performed, which required the engagement of episodic and/or working memory resources. Some studies on tumor patients already described a working memory deficit [39]. Working memory is commonly associated with higher functions; since it enables manipulation of temporarily retain information, for example in language tasks. Inefficient working memory was reported, along with other cognitive deficits, in patients with low grade gliomas [40]. Furthermore, due to a reduced global DMN efficiency, patients may need to activate more areas to perform correctly the task. They probably access episodic memory to generate words and verbs, getting information from a baggage of notions already consolidated. This interpretation would concurrently explain the increase and decrease of DMN activity in the hippocampus and medial prefrontal cortex, respectively.

### Study Limitations

There were a number of limitations in our study. First, the difficulty of the specific task may have influenced DMN pattern. In this regard, it was demonstrated in healthy subjects that DMN could be, to some extent, spatially modulated by sensory and cognitive load [25], [36]. Nonetheless, topical areas in the DMN pattern were reported to be consistently found across a large variety of tasks [1], [3]. As a second point, the analysis method based on ICA could be effective for a global description of the DMN activity [2], [5], but may not be sensitive to changes in connectivity between specific areas included in the network. Another limitation of the study is related to the large variability in terms of tumor grading and location of tumors within the brain, including the difference in the degree of overlap between tumors and DMN. It is logical that a tumor would displace neighboring networks, and that this displacement would be sensitive to the exact location of the tumor. Finally, we did not investigate the relationship between reduced DMN connectivity and behavioral deficits [36], as no behavioral data was collected from our patient population. Given the observed correlation in this preliminary study, the relationship between behavior and DMN connectivity in brain tumor patients should be studied.

On a side note, an evaluation of the degree of overlap between DMN BOLD activation and tumor was hindered by three factors. The first is the reduced number of subjects who presented an overlap between DMN and tumors (one and three for low and high grade tumors, respectively). The second is that connectivity maps are influenced by the statistical cut-offs that are chosen which in turn would modify overlap. The third is that the margins of gliomas are not always definable with MRI. Gliomas have an infiltrating growth, which peripherally may be microscopical, well below the resolution of routine MRI sequences thus requiring DTI and spectroscopy to correctly determine extent. Therefore the study design could not permit the accurate determination of an overlap, if not at the macroscopic level. This overlap in DMN BOLD signal and tumor was not observed in our study population, probably also due to the ICA methods used.

### Conclusions

We assessed modifications induced by different characteristics of cerebral gliomas on the DMN. Our data showed alterations related not only to the local brain injury, but also a global network disruption. The finding that modifications in DMN functional connectivity were related to tumor grading provides additional insight in explaining the relationship between brain lesions and cognitive dysfunction, opening new prospects of neurological intervention.
